# Qualitative exploration of the visual function impairments and impacts on vision-dependent activities of daily living in Retinitis Pigmentosa and Leber Congenital Amaurosis: content validation of the ViSIO-PRO and ViSIO-ObsRO measures

**DOI:** 10.1186/s41687-023-00610-x

**Published:** 2023-07-19

**Authors:** Christine Kay, Isabelle Audo, Christel Naujoks, Claudio Spera, M. Dominik Fischer, Jane Green, Todd Durham, Nicola Williamson, Helena Bradley, Melissa Barclay, Joel Sims, Judit Banhazi, Francesco Patalano

**Affiliations:** 1 Vitreoretinal Associates, Gainesville, FL USA; 2 INSERM, CNRS, Institut de la Vision, CHNO des Quinze-Vingts, National Rare Disease Center REFERET and INSERM-DGOS CIC 1423, Sorbonne Université, 75012 Paris, France; 3 Novartis Pharma AG, Basel, Switzerland; 4 Department of Clinical Neurosciences, University of Oxford, Oxford, UK; 5 Oxford Eye Hospital, Oxford University Hospitals NHS Foundation Trust, Oxford, UK; 6 Centre for Ophthalmology, University Hospital Tuebingen, Tuebingen, Germany; 7 Discipline of Genetics, Faculty of Medicine, Memorial University of Newfoundland, Craig L Dobbin Genetics Research Centre, St. John’s, Canada; 8 Foundation Fighting Blindness, Columbia, MD USA; 9 Adelphi Values Ltd, Cheshire, UK

**Keywords:** Retinitis Pigmentosa, Leber Congenital Amaurosis, Visual function symptoms, Health-related quality of life, Observer-reported outcome, Patient-reported outcome, Clinical outcome assessment, Qualitative research, Content validity

## Abstract

**Background:**

Retinitis Pigmentosa (RP) and Leber Congenital Amaurosis (LCA) are rare inherited retinal degenerative disorders. The associated visual impairments have significant impacts on patients’ vision-dependent activities of daily living (ADL), mobility, and distal health-related quality of life (HRQoL). To adequately capture patient and caregiver perspectives in clinical trials, patient and observer-reported outcome instruments must demonstrate sufficient evidence of content validity in the target population. This study aimed to explore the patient experience of RP/LCA and assess the content validity of the Visual Symptom and Impact Outcomes PRO (ViSIO-PRO) and ObsRO (ViSIO-ObsRO) instruments in RP/LCA.

**Methods:**

A total of 66 qualitative, combined concept elicitation (CE) and cognitive debriefing (CD) interviews were conducted (33 adults, 10 adolescents, 8 children and 15 caregivers of children) in the US, France, Germany, and Canada. Patients had a clinical and genetic diagnosis of RP/LCA from a range of genotypes. CE results were used to further inform the development of a conceptual model and CD interviews assessed the relevance and understanding of the 44-item ViSIO-PRO and 26-item ViSIO-ObsRO instruments. Interviews were conducted across two iterative rounds to allow item modifications.

**Results:**

Findings were consistent across RP/LCA genotypes. Night blindness, reduced peripheral vision, vision in very bright lighting and light/dark adaptation were the most frequently reported visual function symptoms impacting vision-dependent ADL and mobility. Impacts on distal HRQoL domains were also reported. The ViSIO-PRO and ObsRO items were well understood by participants and relevant across genotypes. The instructions, 7-day recall period and response scales were well understood and endorsed. Participant and expert clinician feedback supported modifications to item wording, the addition of six new ViSIO-PRO items and one new ViSIO-ObsRO item, and the removal of one ViSIO-PRO item due to lack of relevance.

**Conclusions:**

Findings support the content validity of the ViSIO-PRO and ViSIO-ObsRO instruments for use across RP/LCA genotypes. Ongoing research to evaluate the psychometric validity of the instruments will support future use of the instruments as efficacy endpoints in clinical trials and in general clinical practice to track disease severity and impact of disease on functioning.

## Background

Retinitis Pigmentosa (RP) and Leber Congenital Amaurosis (LCA) are rare inherited retinal degenerative disorders (IRD), characterised by the loss of rod and cone photoreceptor cells leading to progressive visual function symptoms [1, 2]. Patients typically lose night vision in adolescence, peripheral vision in young adulthood, and central vision in later life [3]. Other visual function impairments include difficulties with dark/light adaptation, colour vision and bright lighting. These visual function symptoms experienced as part of RP/LCA can have a significant impact on patients’ vision-dependent functioning relating to activities of daily living (ADL), mobility, and distal health-related quality of life (HRQoL).

There are multiple gene mutations associated with the autosomal dominant, autosomal recessive or X-linked forms of RP/LCA. Mutations in the *RHO*, *USH2A*, *RPGR* and *RP2* genes are some of the most common, while rarer mutations include *RPE65* and *RLBP1* [4]. There is relatively little published evidence regarding the visual impairments experienced by patients with different RP/LCA genotypes and how these affect patients’ vision-dependent ADL and mobility in terms of the severity and progression of the disease [5].

Green et al. conducted one of the first known studies to explore the patient experience of a specific genotype, *RLBP1* RP*,* via the conduct of qualitative concept elicitation (CE) and cognitive debriefing (CD) interviews [6]. Debriefing of existing patient-reported outcome (PRO) instruments including the National Eye Institute Visual Functioning Questionnaire (NEI VFQ-25) [7, 8], Low Luminance Questionnaire (LLQ) [9] and the Visual Activities Questionnaire (VAQ) [10] in *RLBP1* RP indicated that no single existing instrument provides a comprehensive assessment of all visual impairments and functioning concepts relevant to RP/LCA. The US Food and Drug Administration (FDA) recommend in their PRO guidance [11] and Patient-Focused Drug Development (PFDD) series [12] that PRO and observer-reported outcome (ObsRO) instruments intended for use in clinical trials to support treatment benefit and product label claims, should be informed by direct input from patients. Specifically, it is important that qualitative patient research is conducted to identify concepts of interest to the target population and support the content validity of clinical outcome assessment (COA) measures by demonstrating understanding, comprehension, and relevance to the target population. The existing instruments are not considered fit-for-purpose as outcome measures in RP/LCA as they have not been developed with input from RP/LCA patients and lack content validity in this target population. Similarly, no instruments have been developed to date or deemed appropriate for use in the pediatric RP/LCA population, and there is a lack of studies documenting the pediatric patient experience of RP/LCA from the child or caregiver perspective. Notably, due to the progressive nature of the condition, this is likely to differ from the experience of adolescent and adult patients [2, 3].

As a result of this measurement gap, the Visual Symptom and Impact Outcomes PRO (ViSIO-PRO) and ObsRO (ViSIO-ObsRO) instruments were developed based on findings from the previous qualitative interviews in *RLBP1* RP, a qualitative literature review in broader RP/LCA and input from clinical experts [6, 13–15]. The ViSIO-PRO is designed for completion by adults and adolescents (aged 12 years and above) to assesses visual functioning and the associated impacts on vision-dependent ADL and mobility and distal HRQoL. Items measure the level of difficulty experienced by RP/LCA patients in specific situations or when performing a variety of daily activities that significantly rely on visual function. The ViSIO-ObsRO is designed for completion by caregivers of child patients aged 6–11 years. Items assess visual functioning and impacts on vision-dependent ADL and mobility and distal HRQL by measuring the level of observed difficulty the child experiences as reported by a caregiver. As the ObsRO relies on caregiver observation, fewer items are included to only assess concepts that can be directly observed by caregivers.

The overall objective of the study was to conduct qualitative research with patients and caregivers of RP/LCA patients to better understand the patient experience of visual function symptoms and impacts on vision-dependent ADL and mobility, in order to support the development and evaluate the content validity of the newly developed ViSIO-PRO and ViSIO-ObsRO instruments in accordance with the US FDA PRO guidance for industry and PFDD series [11].

## Methods

### Study design

**Fig. 1:**
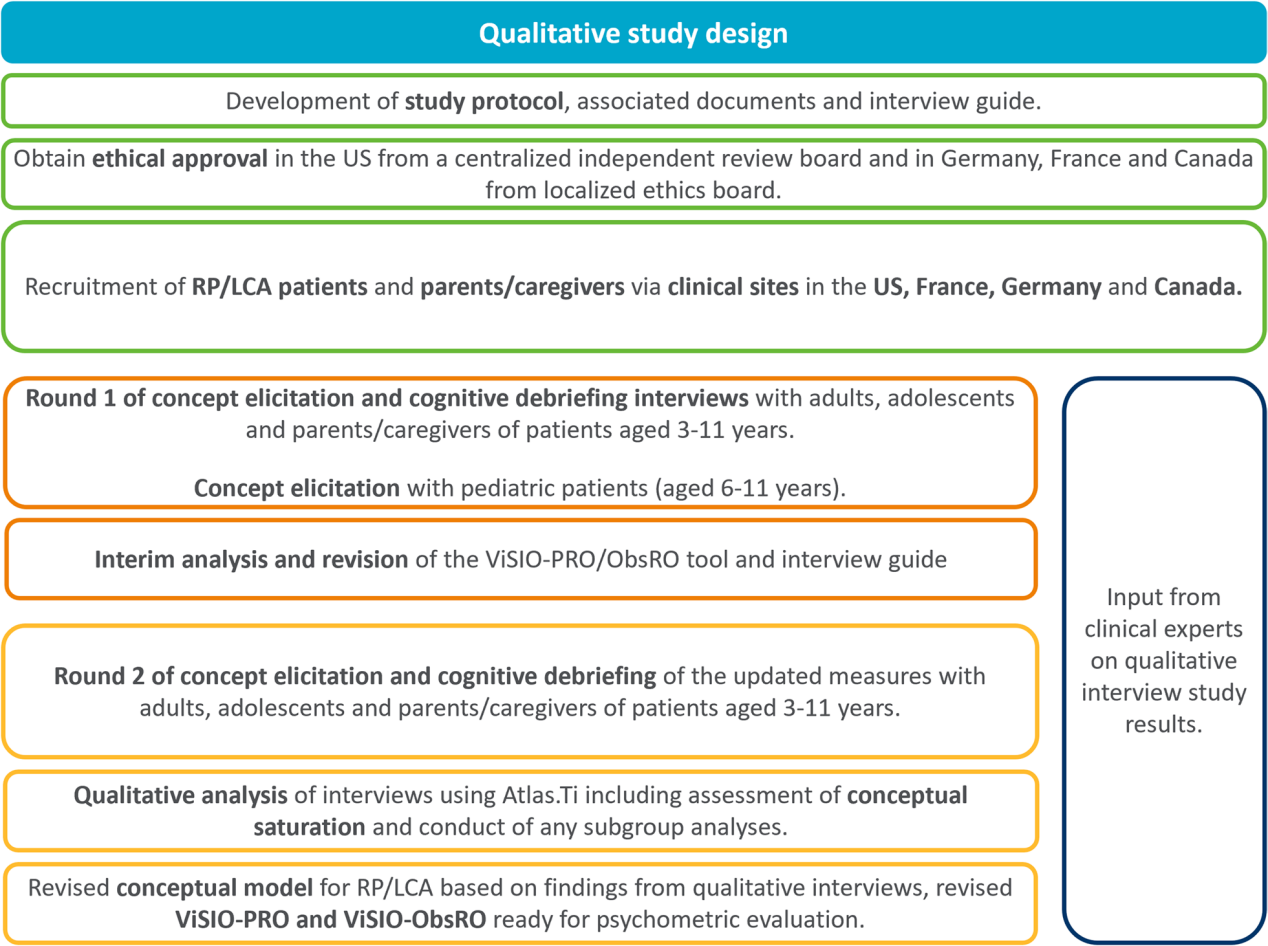
Overview of qualitative study design

This was a qualitative, cross-sectional study involving semi-structured 60-min combined CE and CD interviews with adult, adolescent and child patients with RP/LCA and caregivers of child patients with RP/LCA. The study was approved by a centralised independent review board in the US (approval number: 20190129) and by local ethical review boards in France, Germany, and Canada prior to the conduct of any study-related activities. Figure 1 provides an overview of the study design and processes.

### Recruitment

A sample of 60 participants (patients and parents/caregivers of children aged 3–11 years with RP/LCA) was targeted for inclusion in the study from specialist clinical sites in the US, France, Germany and Canada. Patient participants were required to meet eligibility criteria including: a clinical and molecular diagnosis of RP/LCA confirmed by genetic testing; clinically confirmed visual impairment or significant visual field loss or loss of visual sensitivity, and to be aged 6–11 years (child), 12–17 years (adolescent) or at least 18 years (adult). Caregiver participants were required to be a parent/caregiver of a child aged 3–11 years with RP/LCA. Participants were excluded if they (or their child if a caregiver) had a confirmed diagnosis of Usher Syndrome or any other ocular inherited retina pathologies other than RP or LCA.

The sample size for this qualitative study was defined based on the principles of concept saturation: the point at which no new concept is identified with the conduct of additional interviews [16–20]. Concept saturation can typically be achieved with 12 participants in a homogenous sample. Previous research indicates that 97% of disease-related concepts are usually elicited within 20 interviews and 99% within 25 interviews [21]. Given that patients with different RP/LCA genotypes and ages were targeted for recruitment, a sample of 60 was considered sufficient to achieve concept saturation. Recruitment quotas were employed to ensure the sample had representation across key clinical characteristics (i.e., visual acuity levels, patient/caregiver-reported severity of RP/LCA, and RP/LCA genotype) and participant type (i.e., child, adolescent, adult, and caregiver; Table 1). Targets for specific RP/LCA genotypes were set to ensure representation of the most frequent genotypes in the RP/LCA population [4]. However patients with other genotypes, subject to the eligibility criteria, were also included in the sample to ensure representation of a variety of RP/LCA genotypes beyond those most prevalent in this population.

**Table 1:** Minimum target and achieved recruitment quotas

Criteria	Target (N = 60)	Actual (N = 66)*
Age (years)		
Caregiver of child 3–5 years	n = 1	n = 6
Caregiver of child 6–11 years	n = 1–2	n = 9
Child (6–11 years)	n = 3–4	n = 8
Adolescent (12–17 years)	n = 3–4	n = 10
Adult (18 years and over)	n = 3–4	n = 33
RP genotype		
* RLBP1* RP	n = 1–2	n = 8
* RPE65* RP	n = 1–2	n = 1
* RPE65* LCA	n = 1–2	n = 12
X-linked/*RPGR* RP	n = 1–2	n = 16
* PRPF31* RP	n = 1–2	n = 2
Rhodopsin gene (*RHO*)	n = 1–2	n = 2
Patient-reported severity of RP		
Very mild	n = 1–2	n = 0
Mild	n = 1–2	n = 6
Moderate	n = 1–2	n = 17
Severe	n = 1–2	n = 17
Very severe	n = 1–2	n = 10
Visual acuity* score of the left eye		
Mild (> 60 letters)	n = 1–2	n = 21
Moderate (36–60 letters)	n = 1–2	n = 13
Severe (5–35 letters)	n = 1–2	n = 11
Very severe (< 5 letters)	n = 1–2	n = 10
N/A (blind)	N/A	n = 2
Visual acuity* score of the right eye		
Mild (> 60 letters)	n = 1–2	n = 24
Moderate (36–60 letters)	n = 1–2	n = 13
Severe (5–35 letters)	n = 1–2	n = 10
Very severe (< 5 letters)	n = 1–2	n = 7
N/A (blind)	N/A	n = 2

n = 1 caregiver was included in the total sample twice, as they were interviewed separately about their two children with RP/LCA. For the concept elicitation interviews, counts were included for each child separately however, the caregiver only completed the cognitive debriefing of the ViSIO-ObsRO once

*Visual acuity score as defined by ETDRS (Early Treatment Diabetic Retinopathy Study) letter score (if every letter read correctly on that line and all lines above it on the chart) [26]

Written informed consent (or assent for adolescent and child participants) was obtained prior to the conduct of any study-related activities.

### Data collection

Interviews were conducted in two rounds to allow for modifications between rounds and subsequent testing of the updated instruments. All interviews were conducted by interviewers trained in qualitative interviewing guided by a semi-structured interview guide. Interviews lasted approximately 60 min (20 min CE and 40 min CD).

CE questioning began with broad open-ended questions to spontaneously elicit comments, followed by focused questions to probe on topics of interest. The 44-item ViSIO-PRO and 26-item ViSIO-ObsRO instruments were then cognitively debriefed with adult and adolescent patients and parents/caregivers of children aged 3–11 with RP/LCA, respectively. These instruments include items designed to assess visual function symptoms (i.e., patient/caregiver-reported visual impairments) and the associated impacts on vision-dependent ADL and mobility, and impacts on distal HRQoL (including social functioning, emotional wellbeing, leisure activities and work/education). Adult and adolescent patients were debriefed on either a self-administered or an interviewer-administered version of the ViSIO-PRO, depending on their visual ability to read the instructions, items and response options themselves. Patient and caregiver global impression of severity items (PGI-S and CGI-S, respectively) as well as patient and caregiver global impression of change items (PGI-C and CGI-C, respectively) were also developed and debriefed, to support their use as anchors in future meaningful change analyses for the ViSIO-PRO and ViSIO-ObsRO instruments. The global impression of severity items ask participants to rate the severity of their/their child’s vision problems over the past seven days while the global impression of change items ask participants to rate their/their child’s vision problems over the past 7 days, compared to before they received the study treatment. Each global impression items uses a 5-point verbal descriptor response scale.

The cognitive debriefing involved a ‘think-aloud’ approach whereby participants spoke aloud their thoughts and opinions on the instruments as they completed each item. Participants were also asked detailed questions to explore their understanding and interpretation of the instructions, items, response options and recall period, and relevance of concepts assessed by the instrument. Understanding and relevance of the instructions, items, response options and recall period of the global impression items were also assessed.

### Analysis

All interviews were audio-recorded and transcribed verbatim. Transcripts were qualitatively analysed using Atlas.ti 8 software [22]. Thematic analysis was conducted on the CE portion of the interviews to identify relevant concepts including symptoms, moderating environmental factors, impacts on vision-dependent ADL, mobility and distal HRQoL and coping strategies/visual aids used by patients to cope with their disease and any other relevant concepts [16, 23, 24]. The CD portion of the interviews was also qualitatively analysed to assess participants’ understanding and interpretation of the instructions, items, response options and recall period, and relevance of concepts assessed by the ViSIO-PRO/ObsRO instruments and global impression items.

Based on the interview findings, a conceptual model was developed to display the key concepts associated with RP/LCA, including visual function symptoms, moderating environmental factors, vision-dependent ADL and mobility, impacts on distal HRQoL and coping strategies and visual aids used by patients to cope with their disease. Findings of the CD portion of the interviews were used to guide any modifications to the instruments.

Concept saturation was evaluated by grouping transcripts chronologically into equal sets. Concepts emerging from each additional set of interviews were compared [17, 25]. Saturation analysis was conducted at the participant type (i.e. adult, adolescent, child or parent/caregiver) and total sample level and was deemed achieved when no new concepts emerged in the final set of transcripts.

## Results

### Sample characteristics

A total of 66 participants were interviewed as part of the study, including 33 adults, ten adolescents aged 12–17 years, eight children aged 6–11 years with RP/LCA and 15 parent/caregivers of a child aged 3–11 years with RP/LCA. Participants were recruited from the US (n = 28), France (n = 20), Germany (n = 10) and Canada (n = 8). Table 1 provides an overview of the sample characteristics alongside the corresponding target quotas.

Target quotas were met for age, RP genotype, each of the visual acuity severity scores for the left and right eyes, and most patient-reported severity categories (with the exception of the very mild category for which no patients were recruited).

A range of different RP/LCA genotypes were recruited in the overall sample and all quotas for the six genotypes of interest (i.e., *RLBP1*, *RPE65* RP, *RPE65* LCA, X-linked/*RPGR* RP, *PRPF31* and *RHO*) were either met or exceeded. Other RP/LCA genotypes were also recruited into the study and supported inclusion of a variety of genotypes beyond the most frequent in the RP/LCA population. Overall, *RPGR* RP was the most common genotype included in the sample (n = 16/58, 27.6%), followed by *RPE65* RP/LCA (n = 13/58, 22.4%) and *RLBP1* RP (n = 8/58, 13.8%).

### Concept elicitation results

**Fig. 2:**
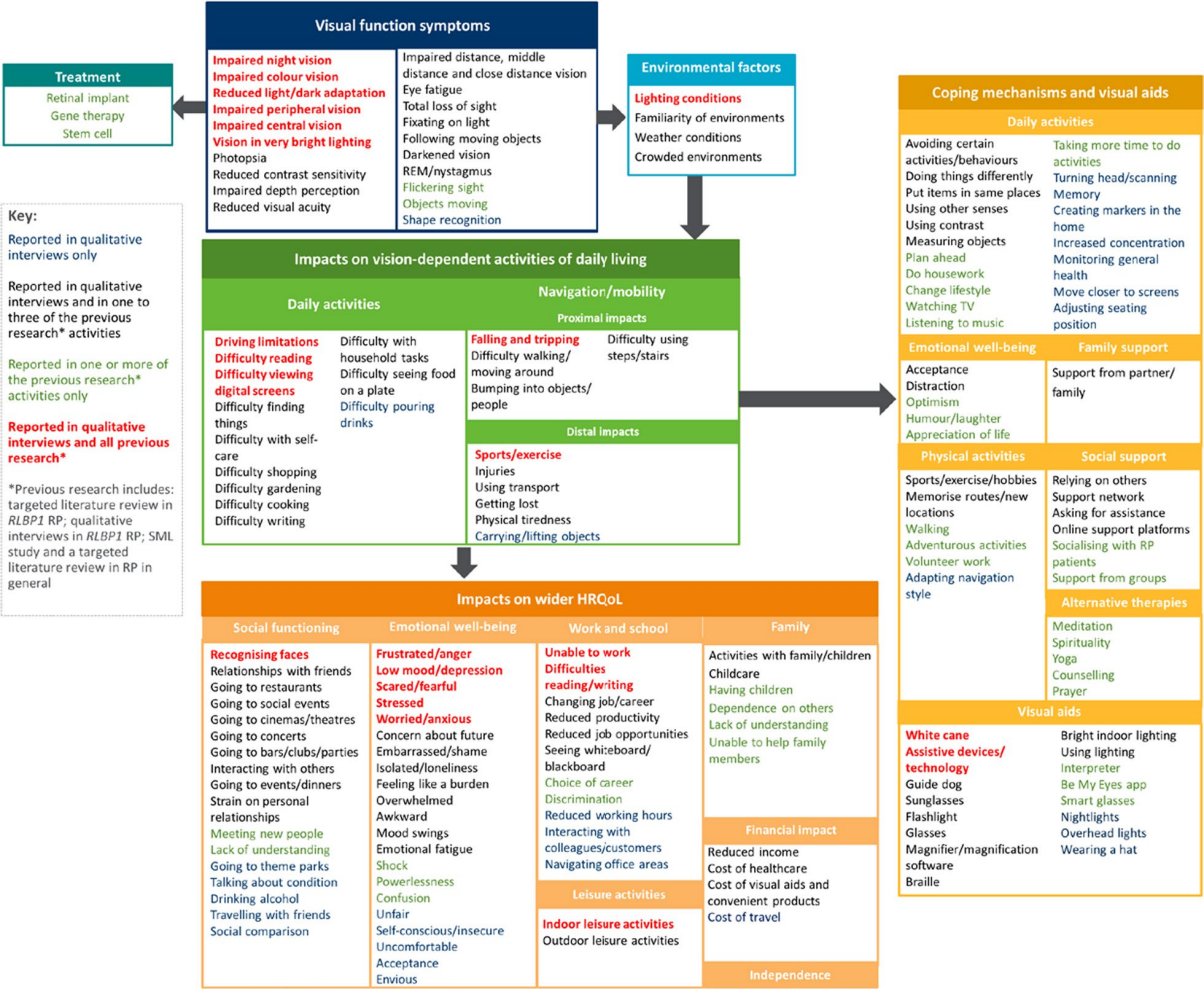
Conceptual model for RP/LCA

The findings of the CE portion of the interviews are summarised in a conceptual model, displaying the key concepts associated with RP/LCA (Fig. 2). The model depicts each of the relevant concepts and the hypothesised relationships among the domains, providing an overview of the broad experience of RP/LCA as reported by the patients and caregivers interviewed and in the literature.

Impacts of visual function symptoms on vision-dependent ADL are grouped as either impacts on ADL or mobility. Impacts on mobility were categorised with regards to their proximity to the condition, with distal impacts more likely to be impacted by other factors and therefore not directly associated with the disease. Impacts on distal HRQoL included impacts on patients’ social functioning, emotional well-being, leisure activities, family relationships, independence, financial situation and work and education.

Table 2 provides an overview of the key symptoms, impacts and environmental factors identified from the CE portion of the interviews alongside supportive quotes. Twenty-two visual function symptom concepts were reported. Key symptoms reported by the majority of participants included night blindness (n = 63), impaired peripheral vision (n = 61), difficulties with vision in very bright lighting (n = 57) and reduced light to dark adaptation (n = 55). These visual function symptoms were also reported in each of the previous research activities (Fig. 2) as described elsewhere [6, 13, 27].

**Table 2:** Overview of key symptoms, impacts and moderating factors reported by participants (N = 66)

Domain/concept (n)		Example quote (participant description)
*Visual function symptoms*		
Night blindness (n = 63)		“*The most important sign, right away, was night vision. Indeed, it was observed very, very early on that he had night blindness, this is what planted suspicion in us.*” (Caregiver of *RPE65* child patient from France)
Impaired peripheral vision (n = 61)		“*So my peripheral vision, so, um, basically it's like tunnel vision, so all I can basically see is what's right in front of me. I have very limited—I can't see nothing on the side of me, so it's basically all central.*” (*RLBP1* adult patient from Canada)
Reduced vision in very bright lighting (n = 57)		“*It can be glaring, again, kind of that painful needling, um, glare feel sensation where I find I'm squinting my eyes, even just in my kitchen looking out through the kitchen window to the bright yard, it's very painful like a headache kind of, um, throbbing eye and outside it's not easy or feeling safe to be able to walk around because of that focus on just trying to not hurt, you know, in the eye.”* (*PRPF31* adult patient from the US)
Reduced light to dark adaptation (n = 55)		“*I am quite dazzled when I walk from a dark room into the light or *vice versa*. When I come from somewhere where there is bright light, whether it is sunlight or whether it is a brightly lit room and I go into the hallway or I come from outside and go into the garage, then it takes me some time to get used to anything. To get used to the light conditions. Either to the very bright light or to the very dark light.*” (*RPGR* adult patient from Germany)
Impaired colour vision (n = 51)		“*I can like barely see the difference from navy blue and black. Like, um, I don't know, I mean, it's hard to tell the difference from like dark colors, like say like this is dark green or black, I just kind of say black, cause I don't know.*” (*RP2* adolescent patient from the US)
Impaired contrast sensitivity (n = 51)		“*Well, sometimes if it like blends in with the floor, I can't really see it, and I might trip over it or run into it…Uh, sometimes when I run in class trying to get something, um, the backpack might be there, or I might not see it, and I run into it and fall.*” (*RPGR* child patient from US)
Impaired distance vision (n = 41)		*“The further away it gets, the more difficult, uh, it gets for me to see.”* (*RLBP1* adult patient from Canada)
*Impacts on vision-dependent activities of daily living*		
Impacts on vision-dependent ADL (n = 66)	Viewing digital screens (n = 49)	“*On the computer, because I use it a lot for work, I have trouble looking at elements when there is a lot of information on the screen. Especially when it’s not really linear or when there is too much of it. Well, that’s what comes to mind.*” (RPE65 adult patient from France)
	Difficulty finding things (n = 41)	*“I asked my family for a headlamp for my birthday two or three years ago. They pooled some money and bought me a high-quality lamp that helps me tremendously when looking for things in the household. If I don't find something, I reach for the headlamp and usually find what I am looking for.”* (*RPGR* adult patient from Germany)
	Difficulty reading (n = 39)	*“Um, even now to the point of cooking, I do most of the cooking, reading the ingredients or the directions on the packaging is a struggle. Um, it’s gotten to the point of just setting the temperature on the stove or on the oven is a struggle to see the numbers.”* (*RPGR* adult patient from US)
Impacts on mobility (n = 66)	Bumping into objects and/or people (n = 45)	“*Like what I might walk into or bump into or fall down. But if I'm with my—if I have my cane with me, I feel a little more safe, but it's still a little like uncomfortable.*” (*RDH12* child patient from US)
	Difficulty walking and moving around (n = 45)	*“But I would be more inclined to wait for a complete clear path of things, so the same would be true for like just crossing your walking areas, where I would pretty much wait until everybody has kind of done their thing, and they seem to be still and where they need to be, and then I'll proceed and go.”* (*PRPF31* adult patient from US)
	Falling/tripping (n = 38)	*“If I'm inside a bright lighting and I go out—open the door to go outside, it's completely dark. Um, I'm completely disoriented as to knowing where I'm—or what—I'm afraid to—if I go outside, to make a misstep and step—fall over steps or stairs or something of that nature.”* (*RLBP1* adult patient from Canada)
	Difficulty using steps/stairs (n = 37)	*“I can’t judge the distance, like if – especially if it’s a slope or the stairs aren’t even, each step, it messes with me. So, I just feel unsteady.”* (*EYS* adult patient from US)
*Impacts on distal HRQoL*		
Social functioning (n = 65)	Relationships with friends (n = 33)	“*For example, they go out in the evening or at night. I do not trust people that much. I do not trust all people. It happened many times when I went out with friends in the evening that I suddenly found myself alone and did not know what to do, because I could not see anything. That is why i avoid going out with others. That makes me a bit sad, because I would also like to go out with my friends at night or in the evening.*” (RPE65 adolescent patient from France)
Impacts on work and school (n = 63)	Difficulty seeing the reading board (n = 15)	“*…in school, um, he has notes where he has to sit upfront to read, uh, the marker board. Um, and he wears glasses to help him see and read and all that kind of stuff.*” (Caregiver of *RPGR* child patient from US)
Emotional wellbeing (n = 61)	Worried/anxious (n = 40)	“*Just worrying about losing all of my vision. I just—yeah. I do. I sometimes—not a lot, but I do worry.*” (RHO adult patient from US)
Financial impacts (n = 20)	Reduced income (n = 15)	“*I would say at this point, um, the only thing that, that financially its affected is, uh, I used to do, uh, PRN, uh, with home health and that was just like extra after my job and I had to quit doing that…*” (PRPF31 adult patient from US)
*Moderating environmental factors*		
Lighting condition (n = 66)	Dim lighting (n = 66)	“*The biggest thing for him is low light situations, and that's really about the only time it is, um, and it's if he has to unexpectedly navigate with low light*” (Caregiver of *RPGR* child patient from US)
	Very bright lighting (n = 60)	“*You know, like, like I always say on a cloudy day, this is my favorite eye type situation because it's not so bright that my eyes are trying to readjust and everything.*” (*EYS* adult patient from US)
Familiarity of environment (n = 58)	Unfamiliar places (n = 54)	“*But when we're at grandma's, he turns on his little lamp. Then he walks around the room a bit more carefully. You can tell he's a little less safe there.*” (Caregiver of *RP2* child patient from Germany)
	Familiar places (n = 49)	“*In my own home, it's not so bad because I know where everything is too.*” (*RLBP1* adult patient from Canada)

Additionally, numerous functional impacts on proximal vision-dependent ADL such as difficulty viewing digital screens (n = 49), difficulty finding things (n = 41), and difficulty reading (n = 39) were identified; and impacts on navigation/mobility such as bumping into objects and/or people (n = 45), difficulty walking and moving around (n = 45), falling/tripping (n = 38) and difficulty using steps/stairs (n = 37). Impacts on distal HRQoL concepts were also identified including impacts on social functioning (n = 65), work and school (n = 63), emotional wellbeing (n = 61) and financial impacts (n = 20). Notably, the visual function symptoms identified in the interviews were consistent across the RP/LCA genotypes included in the sample. Likewise, similar impacts on vision-dependent ADL and mobility, and distal HRQoL were reported across all genotypes.

Participants frequently noted how environmental factors such as lighting condition and familiarity of environment affected the severity of their visual function symptoms and the extent of the impact on ADL and mobility.

### Concept saturation

Saturation was achieved for all visual function symptoms and vision-dependent ADL and mobility and impacts on distal HRQoL when analysed using the total sample. Findings also provided strong evidence that concept saturation had been achieved for most visual function symptoms within each participant type and that symptom concepts had been thoroughly and sufficiently explored.

### Cognitive debriefing of ViSIO-PRO and ViSIO-ObsRO

#### Round 1

Sixteen adult participants and one adolescent participant completed and debriefed the ViSIO-PRO in Round 1. Of these, ten completed the self-administered version and seven completed the interviewer-administered version. The majority of items (n = 38/44) in the ViSIO-PRO were well understood by 82.4% of patients (n = 14/17) without difficulty. The majority of items (n = 38/44) were considered relevant to 70.6% of patients (n = 12/17).

The ViSIO-PRO instructions, including definition of lighting conditions, were broadly understood by most patients who were asked. The recall period was correctly understood and accurately used throughout the ViSIO-PRO by 87.5% of patients (n = 14/16). Similarly, most patients who discussed the frequency and severity response scales understood the direction of the response scales and the response options within these scales. Six patients suggested that the ‘I did not do this at all for reasons other than my vision’ response option should be included in more items.

One caregiver completed and debriefed the ViSIO-ObsRO in Round 1. This caregiver understood all the items that were debriefed and indicated that all of the items discussed would be relevant to their child. Similarly, the ViSIO-ObsRO instructions, recall period and response scales were well understood by the caregiver who debriefed this instrument. Patient and caregiver global impression of severity and change items including the instructions, 7-day recall period and response options were understood by most participants without difficulty.

Based on Round 1 findings and input from clinical experts involved in the care of patients with RP/LCA, some adjustments to the ViSIO-PRO item wording, instructions and response options were made to increase the likelihood of understanding and relevance to this population. In addition, five additional items were added to assess concepts that were reported during the CE interviews that had not been covered by items included in the first version of the ViSIO-PRO. Finally, one item (dressing and bathing) was deleted from the ViSIO-PRO due to lack of relevance.

As the ViSIO-ObsRO was debriefed with one caregiver, no modifications were proposed based on these findings. However, adjustments were made to the item wording and instructions to align with changes recommended to the ViSIO-PRO. Specifically, minor revisions to item wording were made to ten items and minor adjustments to instructions and definition of lighting conditions. These changes resulted in a 48-item ViSIO-PRO and 26-item ViSIO-ObsRO for debriefing in Round 2.

#### Round 2

Seventeen adults and nine adolescents completed and were debriefed on the ViSIO-PRO in Round 2. Of those, ten completed the self-administered version and sixteen completed the interviewer-administered version. Findings indicated that the majority of items (n = 46/48) in the ViSIO-PRO instrument were again well understood by 84.6% of patients (n = 22/26). The majority of ViSIO-PRO items (n = 40/48) were also relevant to 69.2% of patients (n = 18/26).

The ViSIO-ObsRO was debriefed with 13 caregivers in Round 2. All ViSIO-ObsRO items were well understood by 92.3% of caregivers (n = 12/13). Likewise, the majority of ViSIO-ObsRO items (n = 24/26) were considered relevant to their child’s RP/LCA by 61.5% of caregivers (n = 8/13).

The ViSIO-PRO and ViSIO-ObsRO instructions, 7-day recall period and response scales remained well understood by participants in Round 2. Patient and caregiver global impression items including the instructions, recall period and response options were understood by most patients and caregivers.

Notably, participant understanding across both rounds of interviews was consistent between interviewer-administered and self-administered versions of the ViSIO-PRO and ViSIO-ObsRO.

Based on the findings from the concept elicitation interviews, ‘worry about the future’ was identified as an important concept belonging to the emotional domain not yet assessed in the ViSIO-PRO and ViSIO-ObsRO. An additional item was therefore developed to assess ‘worry about the future’ and added to the instruments. No items in the ViSIO-PRO and ViSIO-ObsRO were deleted following Round 2.

Other modifications were made to the ViSIO-PRO and ViSIO-ObsRO to improve item wording. For the ViSIO-PRO, minor changes were made to the wording of 11 items, specifically updating examples of activities provided in the items to be more relevant to the patient experience; updating ‘without help’ to ‘without help from someone else’; specifying the distance for items assessing distance vision and adding ‘because of your vision’ to several items to ensure patients respond based on their vision. No changes were made to the wording of the ViSIO-ObsRO.

This resulted in the 49-item ViSIO-PRO and 27-item ViSIO-ObsRO intended for psychometric testing in subsequent studies.

## Discussion

The overall objective of the study was to explore the patient experience of RP/LCA and evaluate participant understanding and relevance of the newly developed ViSIO-PRO and ViSIO-ObsRO instruments. This is one of the first qualitative studies to comprehensively explore the patient experience of a range of RP/LCA genotypes from both patient and caregiver perspectives in the US, France, Germany and Canada.

### Concept elicitation

Findings from the CE interviews across RP/LCA subtypes provide evidence of a wide range of visual function symptoms that patients experience and the significant impact on patients’ vision-related functioning and distal HRQoL.

The results of this study are supported by findings from the previous literature in *RLBP1* RP, which are consistent with these results and suggest that findings specific to the *RLBP1* RP population can be extrapolated to broader RP/LCA [6, 13]. Key symptoms of *RLBP1* RP such as night blindness, and difficulty adapting to changes in lighting, also had equivalent relevance in the distal RP/LCA sample included within this study. There were little to no apparent differences in the key symptoms (and HRQoL impacts) reported between the specific RP/LCA genotypes included in this study, further supporting the generalisability of these findings.

However, these interviews also identified further visual function symptoms that have not been previously reported in the published literature such as shape recognition which was reported in this study by five *RPE65* and *CEP290* patients/caregivers exclusively. This is likely a specific visual function symptom which may not present as an issue for patients of the *RLBP1* RP sub-type and may not have been previously identified due to the limited depth of information available in the existing qualitative literature. Impacts on vision-related functioning not previously reported were also observed in this study and could be grouped under impacts on daily activities, navigation/mobility, distal HRQoL and coping mechanisms.

Overall, the CE interview findings suggest a high level of consistency across RP/LCA genotypes regarding the symptoms and functional impacts that are relevant to measure as part of future clinical trial programmes.

### Cognitive debriefing

The ViSIO-PRO and ViSIO-ObsRO instruments assess a range of visual impairments and impacts on vision-dependent ADL and mobility ADL and distal HRQoL, which were identified as important aspects of the patient experience of RP/LCA based on prior research [6, 13] and confirmed during the qualitative CE interviews.

The ViSIO-PRO and ViSIO-ObsRO instruments were developed to comprehensively assess the patient experience of RP/LCA, while accounting for the limitations of existing PRO instruments developed in ophthalmology more broadly. These limitations include lack of assessment of concepts in different familiarities of environment and lighting conditions. Additionally, as existing instruments have not been developed specifically for use in RP/LCA they may not necessarily be comprehensive of the patient experience of RP/LCA.

Findings from this study support the content validity of the newly developed ViSIO-PRO and ViSIO-ObsRO instruments and patient/caregiver global impression of severity and change items. The majority of participants understood the ViSIO-PRO and ViSIO-ObsRO instructions, items, response options and recall period well and as intended. Importantly, participants indicated that the concepts assessed by the items were relevant to their (or their child’s) experience of RP/LCA.

Following the first round of interviews, findings informed modifications to the instruments including the addition of five new items to assess vision in fluorescent lighting, middle and long distance vision, depth perception and driving. Additionally, minor changes were made to item wording to improve clarity and understanding for subsequent testing in the second round of interviews. Findings from round two of the interviews indicated that almost all items in the ViSIO-PRO (95.8%, n = 46) were understood by 85.0% (n = 22) of the 26 patients included in round two. There were only two items for which more than four patients (five or six) had difficulty understanding or did not interpret the item as intended; thus even for these two items, most patients still had no difficulty. This included item 6 (seeing in fluorescent lighting) and item 21 (driving in dim lighting). Revisions were implemented for 12 items in the ViSIO-PRO, to promote patient understanding and consistent interpretation of the concepts being assessed. Additionally, a new item was added to assess worry or anxiety about changes to vision in the future. The majority of items were also considered relevant to most patients, with 40 of the 48 items reported as relevant to 69% of patients (n = 18/26). The items that patients did not interpret as intended were also those that lacked relevance to patients. However, it is important to note that some items that lack relevance for less severe patients may be markers of greater severity and therefore important concepts to measure in the instrument, to track changes over time following treatment in the context of a clinical trial.

With regard to the ViSIO-ObsRO, caregivers demonstrated good understanding of items in the round two interviews, with all items understood by 85.0% of caregivers (n = 22/26) and 24 of the 26 items reported as relevant to 62.0% of caregivers (n = 8/13). A proportion of caregivers indicated that some items lacked relevance as they had not observed their child have difficulties with the activities. However, a significant number of caregivers reported the items to be relevant concepts, supporting their retention in the ViSIO-ObsRO. The only modification implemented for the ViSIO-ObsRO instrument was to add a new item to assess caregiver’s perception of their child’s worry or anxiety about changes to their vision in the future, to align with the ViSIO-PRO. These changes led to the 49-item ViSIO-PRO and 27-item ViSIO-ObsRO instruments intended for psychometric validation.

Since the conduct of this research, a new PRO instrument has been developed for use in IRDs more broadly; the Michigan Retinal Degeneration Questionnaire (MRDQ) [28, 29]. The MRDQ is designed to evaluate the treatment benefit of gene therapies across a range of IRDs (cone, cone-rod, rod-cone, or macular dystrophies) from the patient perspective. As such, this instrument is not designed specifically for use in RP/LCA and is largely focused on capturing the visual function symptoms occurring across a range of IRDs. This is in contrast to the ViSIO-PRO and ObsRO instruments which have been developed for use and tested specifically in the RP/LCA population. Additionally, as shown by the current study, a number of vision-dependent ADL and mobility concepts were reported to be significantly impacted within this population, aspects of which are not fully captured by the MRDQ due to its focus on assessing visual function impairments. Therefore, in accordance with regulatory guidance for COA development and validation [11, 12] the ViSIO-PRO and ViSIO-ObsRO instruments have been developed to comprehensively assess the patient experience of the intended target population.

### Study limitations

Findings should be interpreted in light of the limitations of the study. Although an extensive range of RP/LCA genotypes were included in this study, the sample was not exhaustive across all known RP/LCA genotypes. Additionally, the number of patients in both the adult/adolescent sample and children aged 3–11 years sample with certain RP/LCA genotypes was relatively small, making it difficult to draw conclusions about all specific genotypes, although all genotype quotas were met. However, findings suggest that the patient experience of RP/LCA is relatively consistent across genotypes, supporting the relevance of the same concepts to measure across RP/LCA genotypes. Further research in other RP/LCA genotypes is recommended. The majority of participants were recruited from the US (n = 24) and France (n = 20) with fewer participants from Germany (n = 10) and Canada (n = 8). This may potentially limit the generalisability of the study findings to other countries, however findings indicated consistency in the concepts relevant to measure across genotypes and no country differences were identified. Additionally, the patient-reported severity quota for ‘very mild’ was not met in this sample as no patients reported this level of severity. However, all other quotas were met including each of the visual acuity severity scores, which supports that the sample included a range of RP/LCA disease severities and demographic characteristics.

## Conclusion

The results from this qualitative research study provide in-depth insights into the patient experience of RP/LCA across genotypes and provide evidence to support the content validity of the ViSIO-PRO and ViSIO-ObsRO instruments to assess visual function impairments, vision-dependent ADLs and mobility distal HRQoL in RP/LCA, in accordance with the US FDA PRO Guidance (2009) [11] and PFDD guidance [12]. A non-interventional observational study will evaluate the psychometric properties of the ViSIO-PRO and ViSIO-ObsRO instruments and support their use in future RP/LCA clinical trials to inform trial endpoints and ultimately support product label claims, and to track disease severity and impact of disease on functioning in general clinical practice and future research studies.

## Data Availability

The data described in this article are not publicly available in further detail beyond that provided in the manuscript.
